# Recovery when you are on your own: Slow population responses in an isolated marine reserve

**DOI:** 10.1371/journal.pone.0223102

**Published:** 2019-10-10

**Authors:** Jack C. Olson, Richard S. Appeldoorn, Michelle T. Schärer-Umpierre, Juan J. Cruz-Motta

**Affiliations:** 1 Department of Marine Sciences, University of Puerto Rico, Mayagüez, Puerto Rico, United States of America; 2 H.J.R. Reefscaping, Cabo Rojo, Puerto Rico, United States of America; University of Plymouth, UNITED KINGDOM

## Abstract

Geographic isolation is an important yet underappreciated factor affecting marine reserve performance. Isolation, in combination with other factors, may preclude recruit subsidies, thus slowing recovery when base populations are small and causing a mismatch between performance and stakeholder expectations. Mona Island is a small, oceanic island located within a partial biogeographic barrier—44 km from the Puerto Rico shelf. We investigated if Mona Island’s no-take zone (MNTZ), the largest in the U.S. Caribbean, was successful in increasing mean size and density of a suite of snapper and grouper species 14 years after designation. The La Parguera Natural Reserve (LPNR) was chosen for evaluation of temporal trends at a fished location. Despite indications of fishing within the no-take area, a reserve effect at Mona Island was evidenced from increasing mean sizes and densities of some taxa and mean total density 36% greater relative to 2005. However, the largest predatory species remained rare at Mona, preventing meaningful analysis of population trends. In the LPNR, most commercial species (e.g., *Lutjanus synagris*, *Lutjanus apodus*, *Lutjanus mahogoni*) did not change significantly in biomass or abundance, but some (*Ocyurus chrysurus*, *Lachnolaimus maximus*), increased in abundance owing to strong recent recruitment. This study documents slow recovery in the MNTZ that is limited to smaller sized species, highlighting both the need for better compliance and the substantial recovery time required by commercially valuable, coral reef fishes in isolated marine reserves.

## Introduction

Overfishing has fundamentally altered marine coastal ecosystems and currently represents a major source of biodiversity loss [1–3]. Coral reefs have proven particularly susceptible as evidenced by disrupted trophic structures and phase shifts toward algal-dominated conditions [4–5]. Puerto Rico’s coral reefs have not escaped overfishing, the result being depressed spawning stocks [6], reduced total landings [7], and commercial extinction of species [8]. Spatial management schemes, particularly no-take marine protected areas (MPAs), can provide a useful approach for rebuilding and conserving coral reef fish assemblages, and have been implemented to a limited extent in Puerto Rico [9–12]. Long-term monitoring of these sites is necessary to elucidate patterns of recovery and population replenishment in the interest of informed management action [13]. Properly designed and managed no-take MPAs (marine reserves) can bolster fish abundance and species diversity [14–17], protect and increase spawning stock biomass [18–20], increase trophic position of consumer species [21], and perhaps most importantly preserve ecosystem resilience to future disturbance [22]. The success of a marine reserve depends largely upon its size [23,24], age [25,26], isolation from similar benthic habitats [27], and degree of enforcement [28]. Meta-analyses by Edgar et al. [28] found that MPAs which were no-take, well enforced, old (more than 10 years), large (greater than 100 km^2^), and isolated by habitat discontinuities were most effective, but possessing at least four of these characteristics was sufficient to realize substantial gains in fish biomass.

While isolation on a local scale can be beneficial in terms of limiting anthropogenic impacts [29] and stemming emigration of adult fish outside of reserve boundaries [28], isolation on a larger geographic scale can limit the flow of larval recruits, thereby impeding population growth, especially when base populations are small [30–33]. The degree to which protected populations rely upon self-recruitment for persistence depends not only on distance from other larval sources, but also individual species’ larval characteristics and local oceanographic dynamics [34,35]. Marine reserve assessments should consider these factors to provide realistic site and species-specific predictions of recovery [36].

In Puerto Rico, the Mona and Monito Island no-take zone (MNTZ), established in 2004, is the largest NTZ in the U.S. Caribbean [12]. The MNTZ was originally designated with the stated goal of biodiversity conservation but without any objectives specific to fisheries management [37]. The MNTZ now encircles both islands and encompasses coral reef and colonized hard-bottom habitats within a total area of 81 km^2^. Mona Island’s platform is surrounded by deep waters (> 500 m) of the Mona Passage, and lies 44 km distant from the nearest shallow coral habitat [38]. The Mona Passage is recognized as a partial biogeographic barrier limiting the regional distribution and gene flow of some species between the western and eastern Caribbean [39–43]. Several studies have indicated limited population connectivity of reef organisms at Mona with populations in the Dominican Republic [44], or on the Puerto Rican platform [45].

The objective of this study was to assess the effectiveness of the MNTZ by evaluating changes in the biomass and density of a suite of snapper and grouper species 14 years after no-take designation. Most chosen species were heavily harvested pre-closure, while the remaining taxa dominated density trends during initial surveys [46], warranting their inclusion here. All species selected were surveyed in both prior studies (2005 and 2010). The most recent assessment conducted in 2010 found increased densities of small-sized predators after five years of designation [46]. Larger, commercially valuable snapper and grouper species showed no significant reserve effect in either mean biomass or density. The authors mentioned insufficient recovery time, low compliance rates, and spillover of fish into the then-fished areas as potential reasons that a clear reserve effect was not observed. Given that the MNTZ now approximates four of five criteria deemed important for success by Edgar et al. [28], with compliance untested, we assessed whether the prediction of increased biomass from that study has occurred, or whether geographic isolation and small population sizes might be ecological impediments to recovery. With these connectivity characteristics, we expected that, with other factors held constant, recovery of snapper and grouper populations in the MNTZ would be substantially slower than in a less isolated reserve.

With no area currently open to fishing on Mona’s insular platform there was no collocated fished-control site available for direct comparison. Instead, we selected the La Parguera Natural Reserve (LPNR), located on the southwest coast of the main island of Puerto Rico, for the estimation of trends in biomass, density and size distribution at a fished site over a concurrent period. Considering site differences in habitat composition, fish species composition, and degrees of larval connectivity, fish population metrics between the two MPAs were not directly comparable [47]. Rather, we sought trends from the LPNR to better interpret the relative importance of fishing pressure and self-recruitment in shaping the recovery of target species in the MNTZ. We expected the LPNR, despite high rates of exploitation [48], to have higher recruitment rates given its larger size (324 km^2^), and greater degree of larval subsidy from the adjacent areas on the Puerto Rican platform [43,44].

## Materials and methods

### Study sites

**Fig 1:**
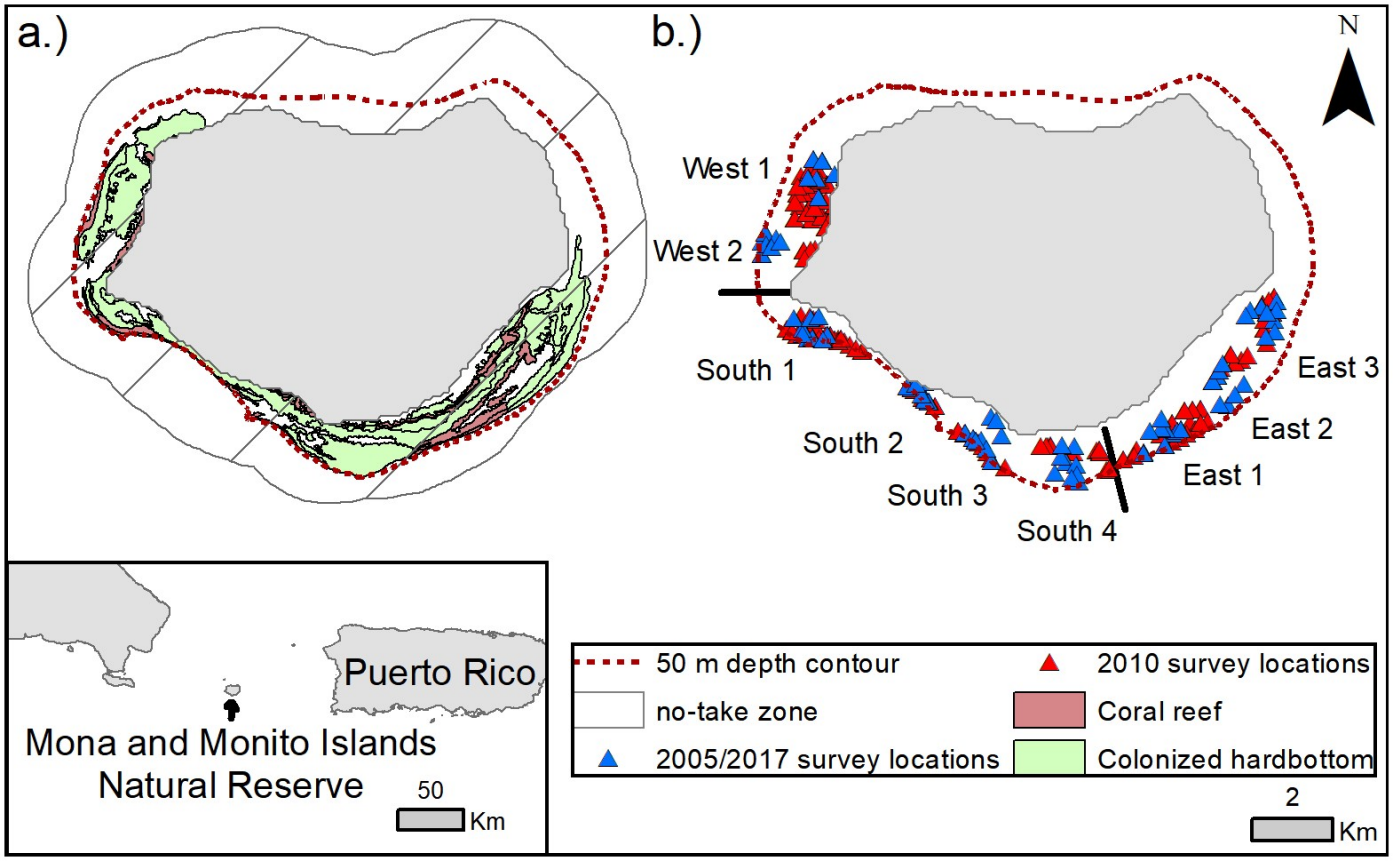
Maps of the Mona Island study area. aCurrent NTZ boundary with coral reef and colonized hardbottom habitat extent, and sampling locations from 2005, 2010, and 2017 (b).

Mona Island is an uplifted carbonate terrace situated to the west of Puerto Rico (18°05'N 67°53'W) in the Mona Passage, a deep strait characterized by strong northward and southward currents [38]. Mona Island is partially encircled on the East, South, and West by a coral reef and colonized hardbottom habitat complex (Fig 1a), has a few, small areas of nearshore seagrass beds, and entirely lacks mangroves [47]. The northern coast is characterized by steeply sloping and deeper hardbottom habitats with little to no coral cover.

Mona’s fringing reefs have historically experienced high fishing pressure, both commercially and recreationally, with concentrated effort directed at grouper spawning aggregations [49]. By 1980, Mona’s Nassau grouper (*Epinephelus striatus*) aggregation had been fished to elimination, and commercial fishing trips to the island slowed in the years thereafter due to declining catches [37]. In 2004, an NTZ was established around most of the island as a special zoning area within the existing MPA. This original design prohibited all fishing activity within 0.5 nautical miles from shore except for a swath on the west coast. In 2007 the boundary of the NTZ was extended to the 100-fathom depth contour to protect multi-species spawning aggregation sites [50], but still excluded habitats off the western coast. Most recently, in 2010, regulations were amended to include the western area in the NTZ resulting in the current 81 km^2^ ring that extends to one nautical mile from shore around both Mona and Monito Islands (Fig 1a).

Without a fished, control site on the Mona insular platform, the LPNR (17°58'N 67°02'W), situated 94 km to the southeast, was chosen for evaluation of temporal trends at a fished location. This area of the Puerto Rico insular shelf extends to 10 km from shore and contains one of the most well-developed, mangrove-seagrass-coral reef complexes in the region [51]. Relative to the MNTZ, fish species composition in the LPNR contains greater densities of mangrove and seagrass-reliant species such as yellowtail snapper (*Ocyurus chrysurus*), mangrove snapper (*Lutjanus griseus*), hogfish (*Lachnolaimus maximus*), and rainbow parrotfish (*Scarus guacamaia*) [47]. The LPNR has never had areas protected from fishing despite attempts to establish spatial fisheries regulations [52]. Historically, fishing pressure in the LPNR has been intense with a variety of gears employed (e.g. traps, gillnets, trammel nets, hook and line, speargun with SCUBA) [53]. Total reported catch in the LPNR declined steeply in recent decades mirroring trends in the number of licensed commercial fishers [54], although increases in the number of recreational anglers has also been noted [55]. A multi-year study of fish assemblage structure and distribution within the LPNR, conducted between 2001–2007, found large decreases in the sighting frequency of large bodied species relative to baseline data collected in 1980 [48]. Nassau grouper sighting frequency declined from 34% in 1980–1981 to only two individuals sighted between 2001 and 2007. Over the course of the later study, however, small grouper (*Cephalopholis fulva* and *C*. *cruentata*) mean biomass and density increased, potentially reflecting indirect trophic effects caused by the prior removal of larger predators.

#### Sampling design and data collection

Data were collected with methods specifically authorized by the Puerto Rico Department of Natural and Environmental Resources (permit number 2017-IC-074). Approval from the University of Puerto Rico Institutional Animal Care and Use Committee (IACUC) was not required due to the purely observational, non-manipulative nature of this project. Fish densities and fork lengths (FL) in both MPAs were estimated via underwater visual census during 10-min, 30 x 2 m transects (60 m^2^). Following each belt transect, a 5-min roving survey was conducted to better detect less abundant, large-bodied species. The area covered in roving surveys was not quantified, but given that surveys were always conducted in similar habitat, with the prevailing current, and limited to 5-min, differences were likely small and unbiased.

In all surveys, divers enumerated snapper and grouper species and estimated fork length (FL) into 5-cm bins. Species counted in 2005 and 2010 surveys of the MNTZ were included in surveys: *Lutjanus mahogoni*, *L*. *apodus*, *L*. *jocu*, *C*. *fulva*, *E*. *guttatus*, *E*. *striatus*, *Mycteroperca venenosa*, and *M*. *tigris*. The list of surveyed species in the LPNR was expanded in 2017 surveys to include *L*. *synagris*, *L*. *griseus*, *L*. *cyanopterus*, *Lachnolaimus maximus*, and boxfishes (Family: *Ostraciidae*) to reflect locally targeted species.

Data collection in the MNTZ followed protocols from the 2005 baseline survey of the reserve [47]. In order to separate areas of differing geomorphology and account for the west coast area that was fished until 2010, the sampling frame was divided into three zones—East, South, and West—containing three, four, and two sites, respectively (Fig 1b). Each 500-m radius site included nine transect locations selected randomly from Schärer-Umpierre’s (2009) sampling points, for a total of 81 transect locations. Sampling sites were separated by at least one kilometer to maintain independence according to published home range sizes of common Caribbean reef fish [56–58]. In the interest of providing greater temporal resolution to the statistical design, data from 108 belt and roving transects conducted in 2010 were assigned to spatially corresponding sites and incorporated in analyses (Table 1; Fig 1b). Transects were limited to hardbottom and coral reef habitats delineated from a habitat map of the Mona insular shelf (minimum mapping unit 100 m^2^) [47].

**Table 1:** Summary of sampling effort.

MPA	Sampling year(s)	Survey method(s)	Seasonal replicates	Total surveys	Depth range (m)	Habitats
**MNTZ**	2005	roving, belt	1	157	5–25	coral reef & col. hardbottom
	2010	roving, belt	1	215	10–20	coral reef & col. hardbottom
	2017	roving, belt	2	312	5–25	coral reef & col. hardbottom
**LPNR**	2004–2006	belt	4	579	5–23	coral reef
	2017	belt	2	219	5–25	coral reef

Summary of survey methods and effort from both MPAs (LPNR and MNTZ) across all study periods.

**Fig 2:**
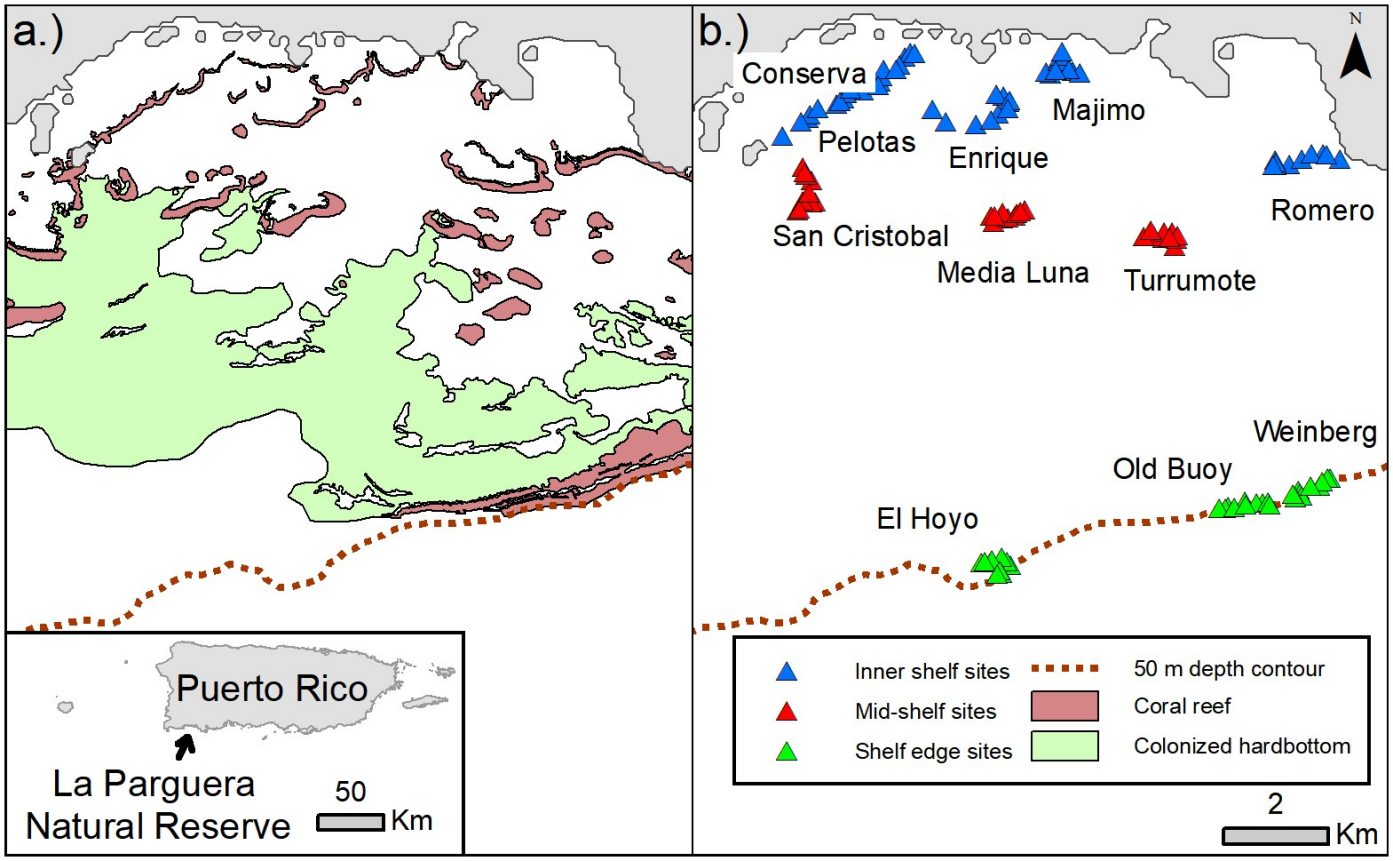
Maps of the LPNR study area. (a) Colonized hardbottom and coral reef habitat extent with 50-meter depth contour and (b) survey sites within each experimental zone (inner-shelf, mid-shelf, shelf-edge).

Sample locations in the LPNR were based upon reef sites with fixed transects sampled previously with four seasonal replicates a year from 2004 through 2006 [59]. Transect sizes differed between sampling periods, with 60 m^2^ transects conducted in 2017 and 100 m^2^ transects surveyed in period 1 (2004–2006). To address this difference, density and biomass in the LPNR were standardized to individuals per meter squared. The experimental design in the LPNR consisted of three zones and 11 sites encompassing 110 total sampling points (Fig 2b). Inner, mid-shelf, and shelf-edge zones respectively contained five, three, and three one-kilometer long (curvilinear distance) sites centered upon original Coral Reef Ecosystem Studies (CRES) program sites [59] and delimited in width and maximum depth by the extent of each forereef. Sites were delineated based upon a 4-m resolution, LiDAR-produced bathymetric map of the region and a habitat map of LPNR (minimum mapping unit of 100 m^2^). Sampling in both the LPNR and MNTZ took place twice in 2017, during spring and winter, to account for potential seasonal effects and to avoid temporal pseudo-replication [60].

#### Statistical analyses

Analyses were conducted on belt transect and roving survey counts and size estimates collected over three time periods (2005, 2010, and 2017) within the MNTZ (Table 1). LPNR analyses were conducted upon data collected during two periods: period 1 encompassing surveys from 2004, 2005, 2006, and period 2 containing 2017 data. Given that roving surveys were not conducted in period 1 in the LPNR, analyses for this MPA were conducted only on belt transect data. Estimated FLs were converted to biomass with species-specific length-weight conversion parameters from Fishbase [61]. No direct comparisons were made between the data collected in the MNTZ and the LPNR due to considerable differences in habitat and species composition [41,47]. Due to the lack of fished and unfished reference areas at Mona and LPNR, respectively, no control sites were assessed. Instead, inferences about the condition of fish assemblages in each location and the performance of the MNTZ were based upon indirect comparison of temporal trends between MPAs.

Differences in biomass and density were assessed with permutational multivariate and univariate analysis of variance (PERMANOVA) in Primer v. 7 [62]. Multivariate tests were conducted on Bray-Curtis dissimilarity matrices of square root-transformed data standardized by sample totals. In the case of multiple, *a posteriori* pairwise comparisons, Bonferroni corrections were applied. In some cases, dummy variables were added prior to matrix construction to allow for the calculation of dissimilarities between roving samples that returned zero fish [63]. Similarity percentage analysis (SIMPER) was applied to identify species contributing most towards the significant multivariate differences; species contributing 30% or more of cumulative between-group dissimilarities were selected for univariate analysis. Univariate PERMANOVA were performed on Euclidean distance matrices of mean total density, mean total biomass, as well as the mean density and biomass of species contributing most to multivariate trends. Only species with samples size of at least 80 individuals were included in this analysis in order to better control for precision and accuracy of estimated population parameters [64]. In cases of heterogeneous variance, a corrective transformation was applied (square root or fourth root for densities, and *log(x+1)* for biomass). If heterogeneity remained after transformation, analysis was conducted on untransformed data, but results were evaluated at the more conservative probability level of *p* < 0.01 [65]. Size distributions of species that contributed most to multivariate differences in each MPA were tested between periods to help interpret univariate trends. In the case of the MNTZ, species’ length data were pooled from roving and belt surveys unless significant differences were found between methods (Mann-Whitney U test) in which case distributions were tested separately by survey method. The shape of length frequency distributions between periods were compared with the two-sample Kolmogorov-Smirnov test and relative locations of each distribution were tested with the Mann-Whitney U test on ranks.

## Results

### Mona Island NTZ

**Fig 3:**
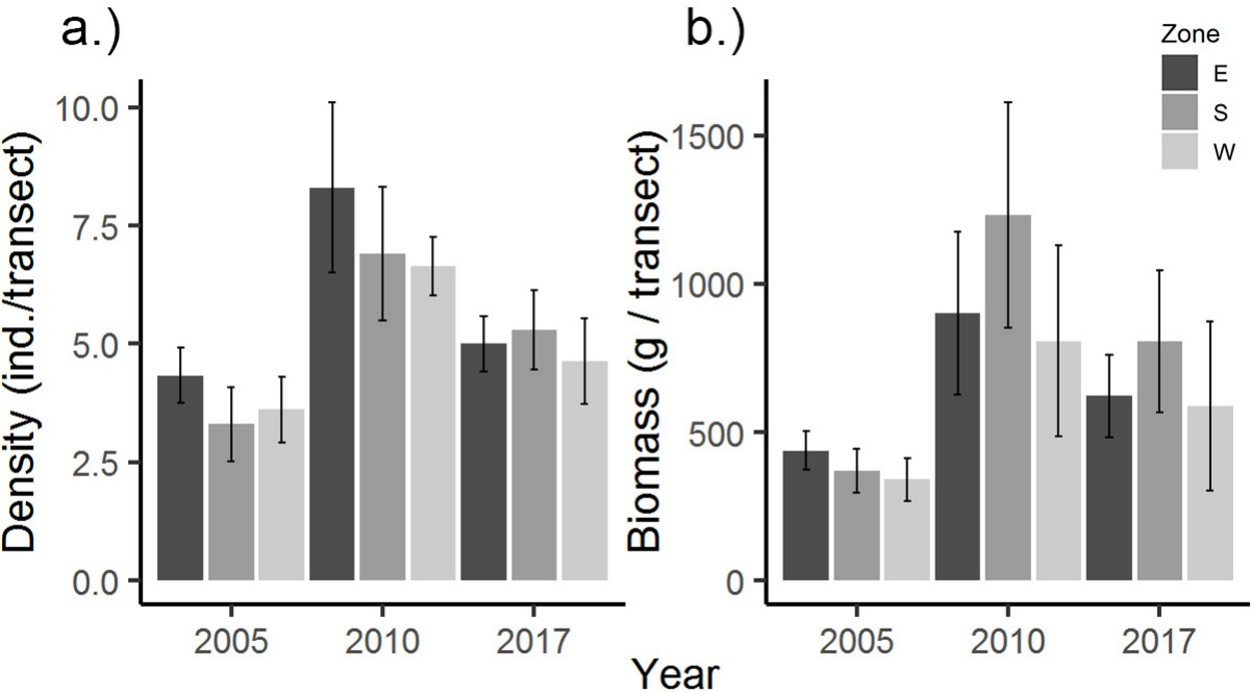
Density and biomass trends from the MNTZ by year and zone. Mean total transect density (a) (fish / 60 m^2^ transect) and biomass (b) (grams / 60 m^2^ transect) by year and zone from the MNTZ, with standard error bars indicated. Level of significance: *p < 0.05; **p < 0.01; ***p < 0.001.

**Fig 4:**
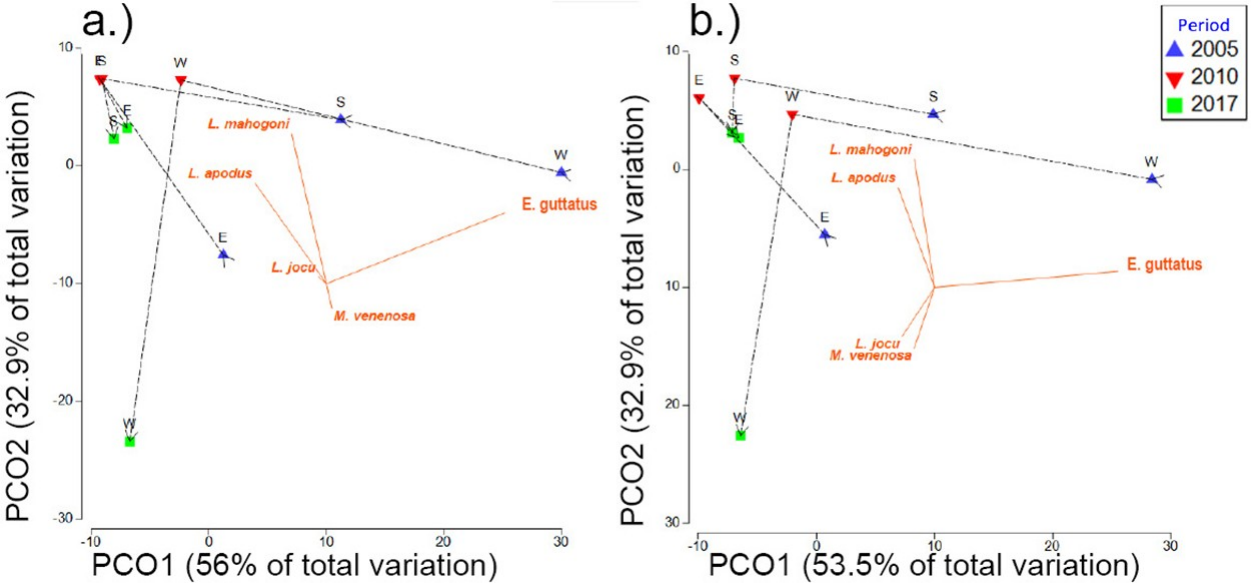
PCO plots of roving density and biomass from the MNTZ. Ordination based upon Bray-Curtis dissimilarities of square-root-transformed density (a) (fish/survey) and biomass (grams/survey) (b) from roving surveys, showing centroids by zone and period overlain with species correlation vectors (Pearson > 0.2).

Univariate PERMANOVA of total combined snapper and grouper biomass and density from the MNTZ found no significant differences between periods except for densities from transects, where all pairwise tests between periods were significant (Table 2). These responses corresponded to a 75–117% (mean ± SE) increase in total density between 2005 and 2010, a 25–35% decrease between 2010 and 2017, and an overall 27–45% increase from 2005 to 2017 (Fig 3). Multivariate PERMANOVA returned significant main effects by period in the densities of species surveyed in belt transects and roving counts (Table 3a and 3b). There was also a significant effect by period on roving biomass, but not transect biomass. Pairwise tests showed significant differences in multivariate transect density between 2010 and the other two survey periods, while multivariate roving density and biomass differed only between 2005 and 2017 (Tables 2 and 3). Contributions to roving survey dissimilarity between periods were greatest from schoolmaster (*Lutjanus apodus*) and red hind (*Epinephelus guttatus*), with red hind driving period-wise separation in the west zone, and schoolmaster contributing most to dissimilarities in south and east zones (Fig 4a and 4b).

**Table 2:** Results of A posteriori pairwise comparisons from the MNTZ.

Comparison	Multivariate								Univariate							
	Transects				Roving surveys				Transects				Roving surveys			
	Density		Biomass		Density		Biomass		Density		Biomass		Density		Biomass	
	*t*	*p*^†^	*t*	*p*^†^	*t*	*p*^†^	*t*	*p*^†^	*t*	*p*^†^	*t*	*p*^†^	*t*	*p*^†^	*t*	*p*^†^
2005, 2010	3.67	**0.003**	1.46	0.154	1.93	0.041	1.74	0.054	5.33	**0.002**	1.52	0.181	0.91	0.411	0.50	0.788
2005, 2017	1.79	0.048	0.84	0.740	2.28	**0.013**	2.11	**0.029**	4.12	**0.003**	1.13	0.400	1.73	0.128	1.48	0.180
2010, 2017	2.14	**0.019**	1.21	0.254	1.68	0.096	1.43	0.180	3.66	**0.005**	1.13	0.397	0.92	0.580	1.62	0.117

Results of pairwise PERMANOVA of univariate and multivariate density and biomass by period from the MNTZ.

^†^ Significant values in bold according to Bonferroni-corrected alpha

**Table 3:** Summary of multivariate PERMANOVA results of snapper and grouper biomass and density from the MNTZ.

**a.) Transects**	**Density**				**Biomass**			
Source of variation^b^	*df*	*MS*	Pseudo-*F*^a^	Unique perms	*df*.	*MS*	Pseudo-*F*^a^	Unique perms
Period	2	3646.3	5.10**	9950	2	2166.4	1.07	9944
Zone	2	5095.8	2.43	9945	2	6830.2	1.44	9935
Zo x Pe	4	1332.5	1.28	9943	4	1774.9	0.81	9921
Residual	313	727.68			313	1934.5		
Total	349				349			
**b.) Roving surveys**	**Density**				**Biomass**			
Source of variation^b^	*df*.	*MS*	Pseudo-*F*^a^	Unique perms	*df*.	*MS*	Pseudo-*F*^a^	Unique perms
Period	2	16375	4.05 **	9949	2	13413	3.30 *	9956
Zone	6	5688.1	4.58 **	9939	2	5925.8	0.87	9949
Zo x Pe	4	5888.7	1.20	9929	4	5267.8	1.07	9942
Residual	298	3215.7			298	3192.7		
Total	333				333			

Tests conducted on Bray-Curtis dissimilarities of root-transformed biomass and density from belt transects (a), and roving surveys (b), conducted in the MNTZ in 2005, 2010, and 2017.

^a^ Level of significance:

* *p* < 0.05;

** *p* < 0.01

^b^Zo = Zone; Pe = Period

Univariate testing of species-specific metrics revealed a significant, 45–76% (mean ± SE) decrease in the mean density of red hind (*E*. *guttatus*) in roving transects between 2005 and 2017. No significant differences were detected between 2005 and 2010, 2010 and 2017, or in mean total biomass of red hind between any period. Coney (*C*. *fulva*) density increased significantly by 28–54% between 2005 and 2010, and 33–51% between 2005 and 2017 (Table 4). The largest surveyed species (*Epinephelus striatus*, *Mycteroperca venenosa*, *M*. *tigris*, and *Lutjanus jocu*) were sighted too infrequently across periods for individual analyses (n < 80).

**Table 4:** Summary of species-specific pairwise tests (MNTZ).

Transects	Species	Pseudo-*F*^a^ (period)	Levene’s test (*F* ^a^)	Transformation
**Density**	*C*. *fulva*	7.69 **	2.92	√
	*L*. *apodus*	1.51	30.21***	none
	*L*. *mahogoni*	0.64	1.36	none
**Biomass**	*C*. *fulva*	0.51	11.88***	log(x+1)
	*L*. *apodus*	0.56	10.02***	none
	*L*. *mahogoni*	1.01	5.13**	none
**Roving surveys**				
**Density**	*E*. *guttatus*	4.01	50.17***	none
	*L*. *apodus*	0.49	0.94	none
	*L*. *mahogoni*	0.58	2.14	none
**Biomass**	*E*. *guttatus*	0.43	9.92	√
	*L*. *apodus*	0.54	0.85	none
	*L*. *mahogoni*	0.63	5.57**	none

Pseudo-*F* values shown from pairwise PERMANOVA of individual species biomass and density between 2005 and 2017 in the MNTZ.

^a^ Level of significance:

**p < 0.01;

***p < 0.001.

### La Parguera Natural Reserve

**Fig 5:**
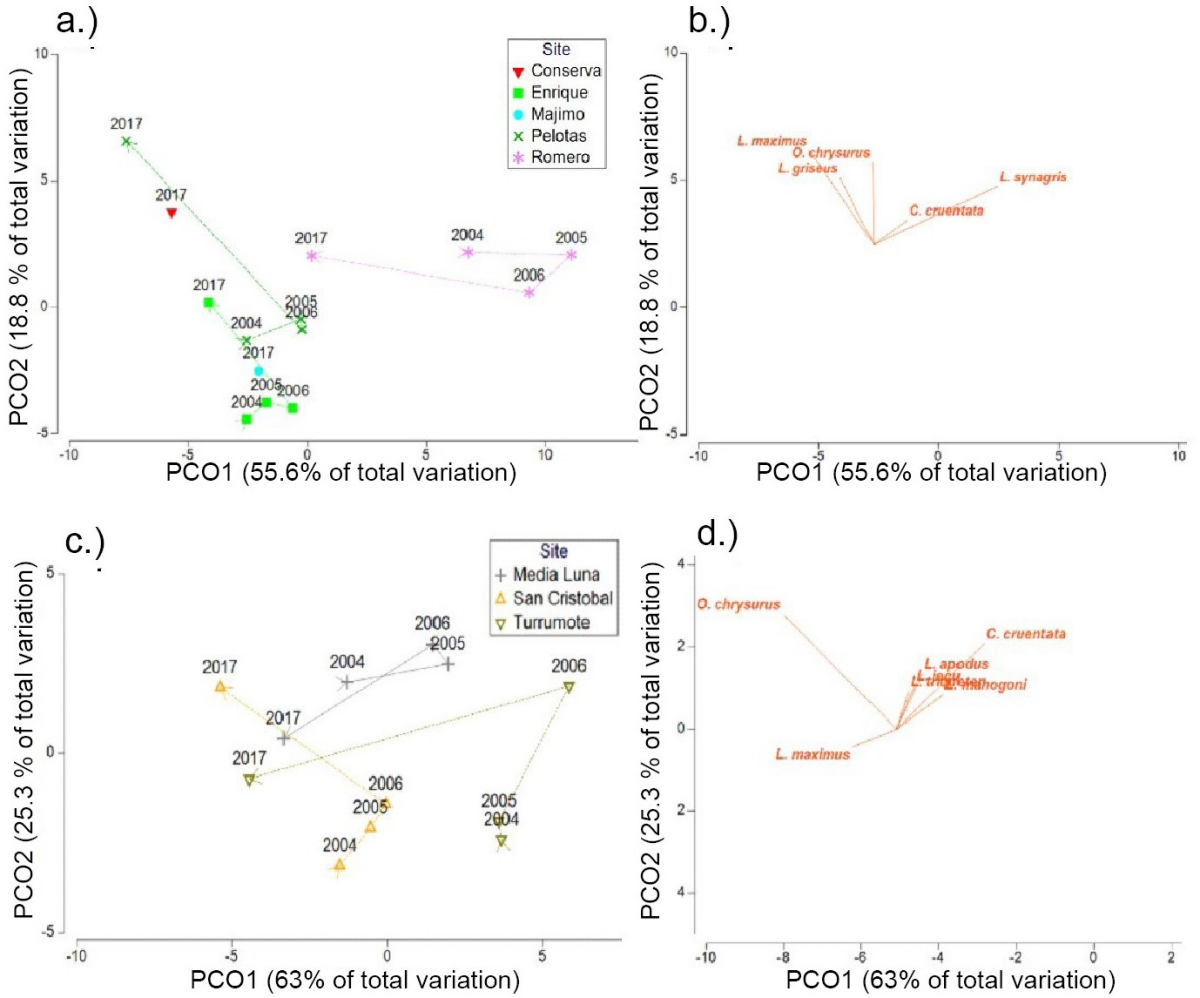
PCO plots showing density trajectories of the LPNR commercial assemblage from inshore and mid-shelf zones. Ordinations showing centroids of root-transformed commercial species density (fish/m^2^) from LPNR by year from inshore sites (a) and mid-shelf sites (c). Adjacent plots (b, d) show corresponding response vectors (Pearson > 0.2) from top contributing species indicating direction and magnitude of contributions to dissimilarities between periods (2004–2006, 2017).

**Fig 6:**
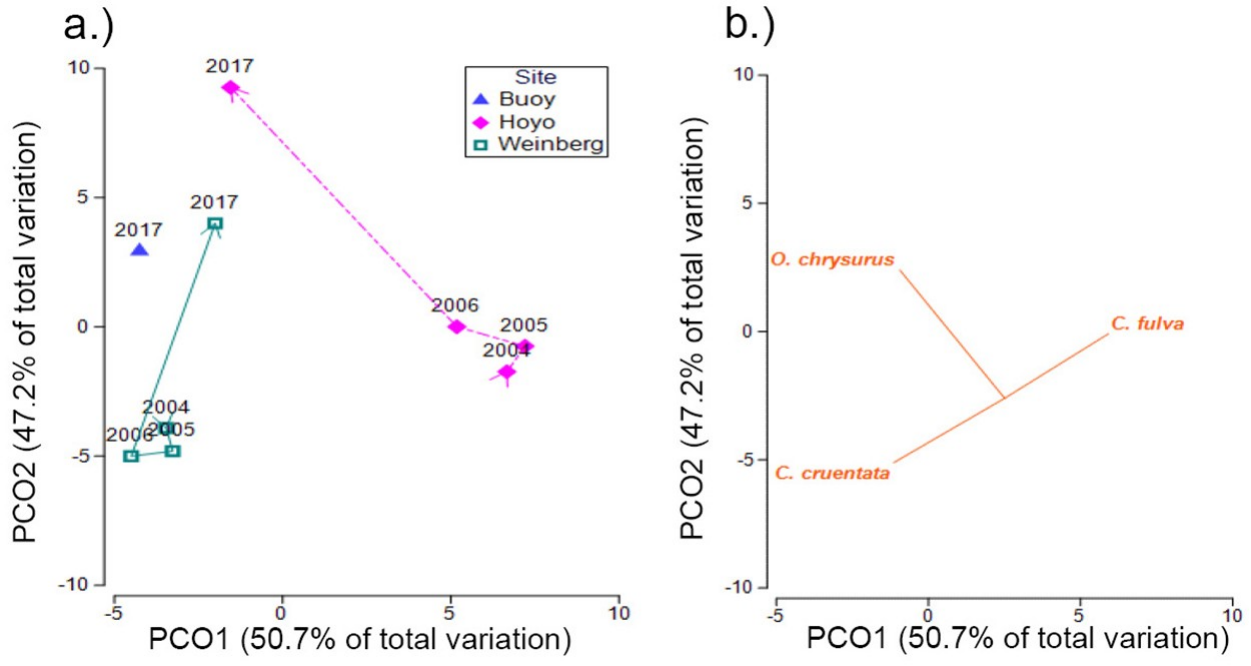
PCO plot of commercial species density from the LPNR shelf-edge zone. Centroids of root-transformed commercial species density from the LPNR by year from shelf-edge sites (a), with species response vectors indicating direction and magnitude of top species’ contributions to dissimilarities (Pearson > 0.2) (b).

**Fig 7:**
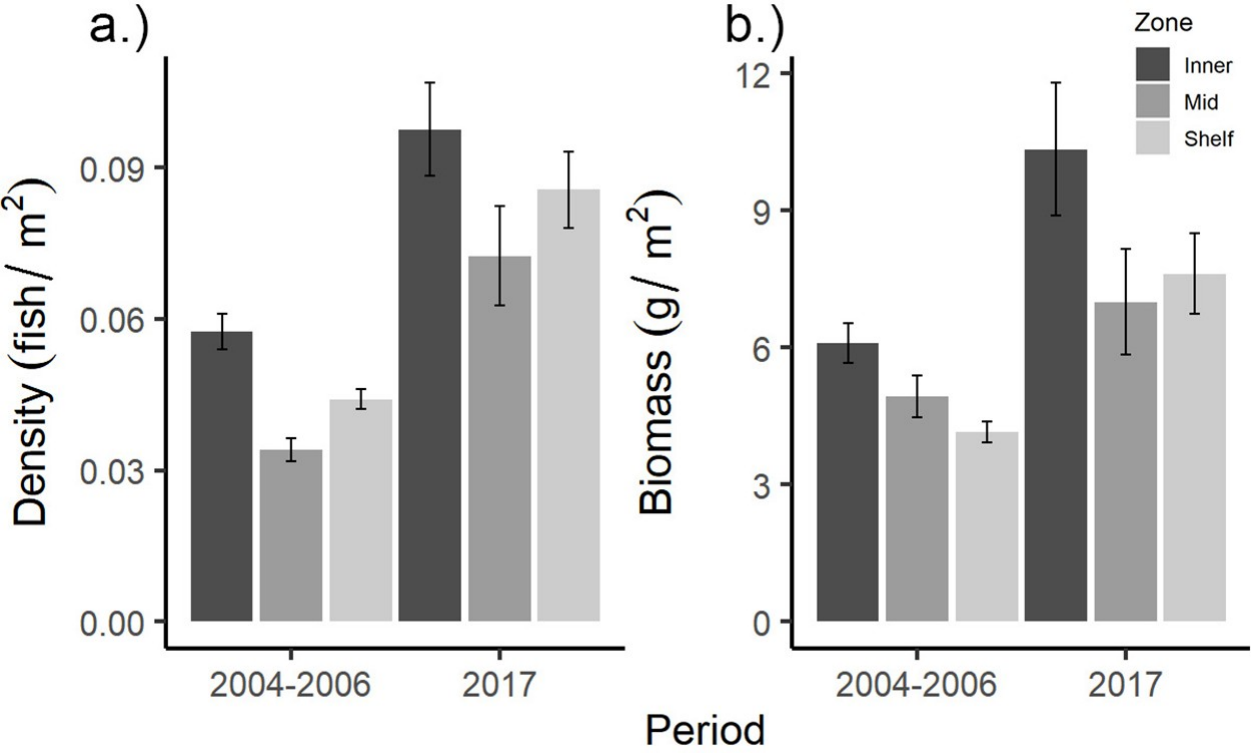
Total density and biomass trends in the LPNR. Commercial species mean density (fish/m^2^) (a), and biomass (g/m^2^) (b) from the LPNR by sampling period (2004–2006, 2017) and cross-shelf zone with standard error margins indicated. Level of significance for period-wise comparisons: * p < 0.05; ** p < 0.01; ***p < 0.001.

A significant multivariate interaction was detected between factors ‘site’ and ‘period’ from the LPNR (Table 5), with all but one reef site (Romero) differing significantly in multivariate density between periods (Table 6). A significant change in multivariate biomass between periods was also detected at 10 of 11 reef sites. SIMPER analysis indicated that yellowtail snapper (*O*. *chrysurus)* and hogfish (*L*. *maximus)* contributed most to density dissimilarities between periods within the inner-shelf zone, with 22% and 13% average dissimilarity, respectively (Fig 5b). Among mid-shelf sites, yellowtail snapper contributed nearly half (46%) of the total dissimilarity in densities between periods, with the greatest overall changes coming from San Cristobal reef (Fig 5c and 5d). As with nearshore zones, at the shelf edge, yellowtail snapper contributed the most (34%) to aggregate density dissimilarity between periods, with an associated mean increase (Table 7, Fig 6). Pooling all species and sites, there was significant 75–113% increase in mean density between periods in the LPNR (Table 8; Fig 7). Four reef sites—Enrique, Pelotas, El Hoyo, and San Cristobal—drove total density gains (Table 8). Biomass also increased at these sites, except for Pelotas reef, while no other reef site produced significant trends in mean biomass between periods.

**Table 5:** Summary of multivariate PERMANOVA (LPNR).

Source of variation ^b^	Density			Biomass		
	*df*	*MS*	Pseudo-*F*^a^	*df*	*MS*	Pseudo-*F* ^a^
Period	1	634.88	12.12 ***	1	1280.20	6.49 ***
Year (Pe)	2	18.02	1.17	2	21.47	1.22
Zone	2	276.87	3.24 **	2	240.44	1.07
Zo x Pe	2	61.93	1.65	2	148.19	0.88
Zo x Ye (Pe)	4	9.88	0.83	4	37.58	1.40 *
Residual	690	9.12		690	45.73	
Total	798			798		

Results of multivariate PERMANOVA of commercial species density (fish/m^2^) and biomass (g / m^2^) from belt transects conducted within the LPNR.

^a^ Level of significance:

*p < 0.05;

**p < 0.01;

***p < 0.001.

^b^ Zo = Zone, Pe = Period, Ye = Year

**Table 6:** Summary of pairwise PERMANOVA by reef site in the LPNR.

Zone	Reef site	Density Pseudo-*F*^a^	Biomass Pseudo- *F*^a^
Inner	Pelotas	11.33 *	7.37 *
	Enrique	16.39 *	15.04 *
	Romero	2.56	5.08 *
Mid	Turrumote	15.97 *	2.63 *
	Media Luna	5.47 **	2.95 *
	San Cristobal	32.59 **	7.14 *
Shelf-edge	Weinberg	15.06 **	11.39 *
	El Hoyo	45.98 **	51.12 *

Comparisons conducted between periods (2004–2006 vs. 2017) on root-transformed density and biomass.

^a^ Level of significance:

*p < 0.05;

**p < 0.01

**Table 7:** Summary of species-specific pairwise tests of density and biomass (LPNR).

	Species	Pseudo-*F* ^a^ (period)	Mean change (%)^b^	Levene’s test *F*^a^ (period)	Transformation
Density	*O*. *chrysurus*	14.54 ***	192%	0.02	√ √
	*C*. *cruentata*	1.50	--	111.64 ***	none
	*L*. *maximus*	6.92 **	580%	413.37 ***	√ √
	*L*. *apodus*	2.21	--	45.08 ***	√ √
	*C*. *fulva*	3.73 *	60%	4.49 *	√
	*L*. *synagris*	0.82	--	48.79 ***	none
	*L*. *mahogoni*	0.77	--	0.10	none
Biomass	*O*. *chrysurus*	13.32 ***	151%	237.14 ***	log(x+1)
	*C*. *cruentata*	2.91	--	314.72 ***	none
	*L*. *maximus*	6.22 **	345%	307.69 ***	none
	*L*. *apodus*	1.19	--	0.162	none
	*C*. *fulva*	0.62	--	2.19	none
	*L*. *synagris*	0.97	--	63.30 ***	log(x+1)
	*L*. *mahogoni*	0.74	--	18.92 ***	none

Results of species-specific univariate PERMANOVA of density (fish/m^2^) and biomass (g/m^2^) between sampling periods (2004–2006, 2017) in the LPNR.

^a^ Level of significance:

*p < 0.05;

**p < 0.01;

***p < 0.001.

^b^—indicates no change

**Table 8:** Summary of univariate PERMANOVA results of total density (fish/m^2^) and biomass (g/m^2^) of commercial species in the LPNR.

Source of variation ^b^	*df*	Total density (Pseudo—*F*^a^ *)*	Total biomass (Pseudo—*F*^a^*)*
Period	1	3.39 *	2.97
Year (Pe)	2	0.84	2.44
Zone	2	1.51	0.48
Zo x Pe	2	0.26	0.43
Si (Zo) x Pe	5	5.25 **	5.46 **
Enrique (Pe. 1 vs Pe. 2)	1	3.18 *	3.74 *
Pelotas (Pe 1 vs Pe. 2)	1	3.65 *	2.89
El Hoyo (Pe. 1 vs Pe. 2)	1	2.88 *	3.21 *
San Crist. (Pe. 1 vs Pe. 2)	1	3.56 **	3.06 *
Zo x Ye (Pe)	4	0.92	2.98
Residuals	690		
Total	798		
Transformation		√ √	log(x+1)

^a^ Level of significance:

*p < 0.05;

**p < 0.01.

^b^ Zo = Zone, Pe = Period, Ye = Year, Si = Site

Species-specific univariate tests of top contributing species biomass and density revealed significant, period-scale differences for yellowtail snapper, hogfish, and coney (Table 8). Across the LPNR survey domain, yellowtail snapper density increased 150–239% (mean ± SE) and biomass increased 117–189%, with the largest gains in both metrics found at San Cristobal (mid-shelf zone)(Table 8). Hogfish mean total density increased by 405–807%, and mean total biomass by 215–523%, while coney increased only in mean total density by a margin of 10–126% (Table 8). Hogfish mean density gains were strongest at Pelotas reef (inshore zone), while coney mean density increased most at Weinberg (shelf-edge zone).

#### Size distributions: Mona NTZ

**Fig 8:**
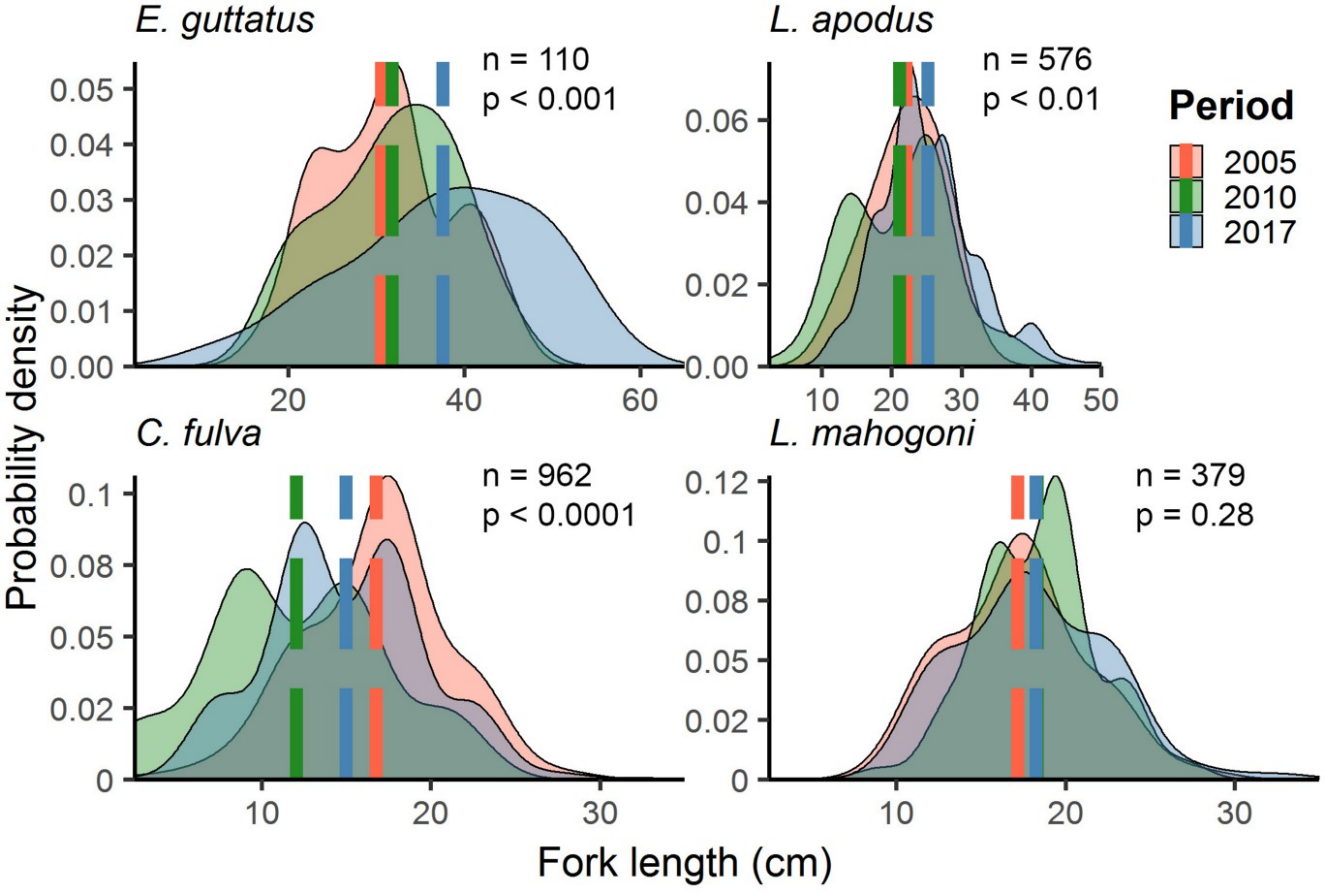
Probability density distributions of estimated fork length from the MNTZ. Dashed lines represent yearly means for red hind (*E*. *guttatus)*, schoolmaster (*L*. *apodus*), coney (*C*. *fulva*), and mahogany snapper *(L*. *mahogoni*). *P* values from Mann-Whitney U tests of mean rank size between 2005 and 2017.

Four species sampled within the MNTZ and seven species sampled within LPNR were selected for length frequency distribution analysis based on a minimum sample size of 80 individuals. In the MNTZ, length distributions differed significantly in location and shape between baseline and current surveys (2005 vs. 2017) for three of four taxa (Table 9, Fig 8). Red hind was the only species assessed for which length estimates differed significantly by survey method across periods, therefore analyses of size distributions for the species were separated between roving and belt transects. Red hind FL collected in roving surveys between 2005 and 2017 increased 10–33% (mean ± SE), while those from belt transects rose 45–98% (mean ± SE). Coney was the only species assessed from the MNTZ that decreased in mean FL between 2005 and 2017, from 16.79 cm (± 0.33 cm) to 14.99 cm (±0.26 cm).

**Table 9:** Results of size distribution tests for the MNTZ.

Species	KS test	Mann-Whitney U
*E*. *guttatus*	*p* < 0.01	*p* < 0.001
*L*. *apodus*	*p* < 0.05	*p* < 0.01
*C*. *fulva*	*p* < 0.0001	*p* < 0.0001
*L*. *mahogoni*	*p* = 0.41	*p* = 0.10

Resulting *p*-values from two-sample Kolmogorov-Smirnov (KS) tests of the equality of size distributions, and Mann-Whitney U tests for location effects in species FL distributions from the MNTZ. Comparisons conducted between 2005 and 2017 data.

#### Size distributions: LPNR

**Fig 9:**
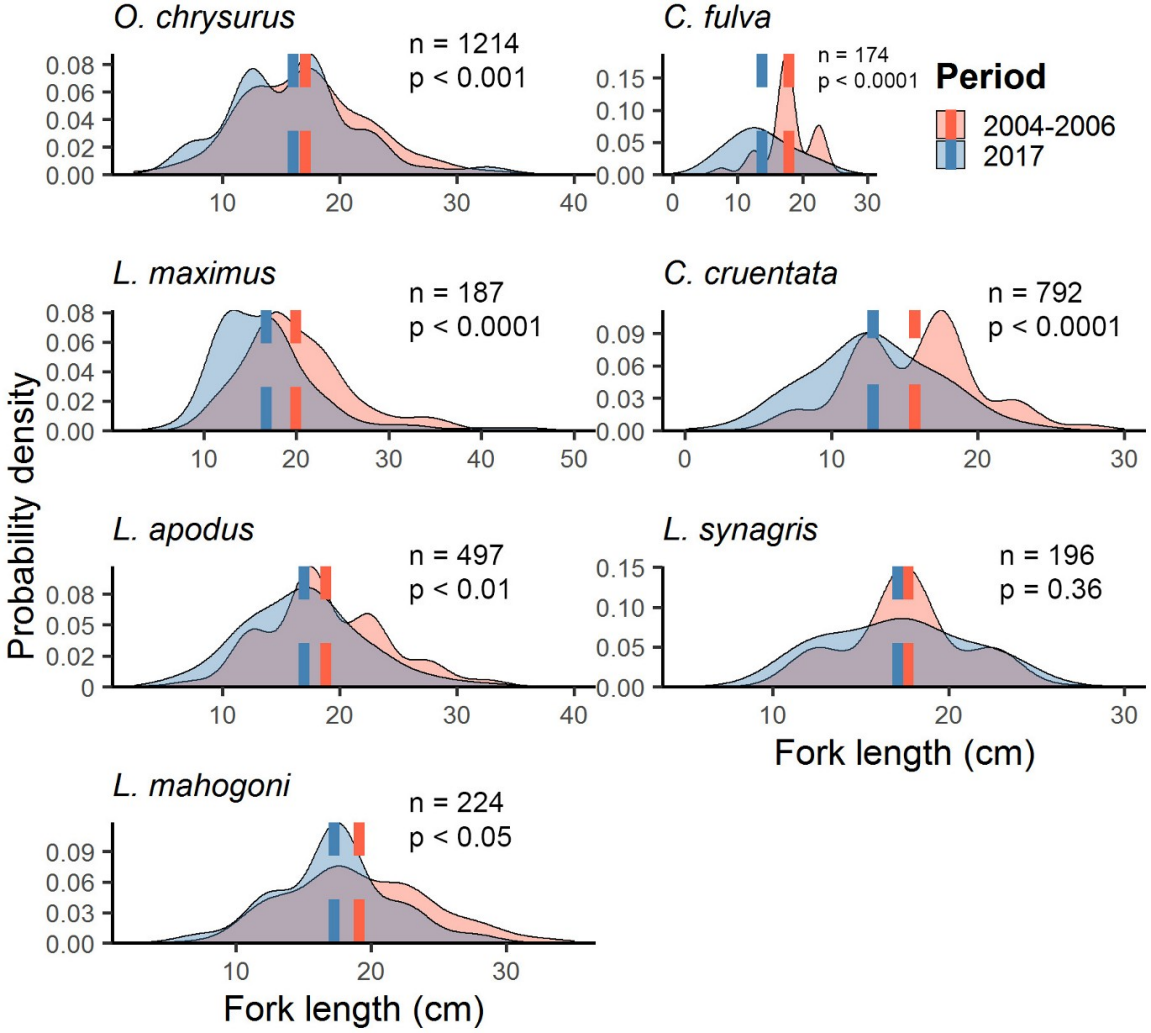
Probability density distributions of estimated fork length of commercial species from the LPNR. Mean fork length for yellowtail snapper (*O*. *chrysurus*), coney (*C*. *fulva*), hogfish (*L*. *maximus*), graysby (*C*. *cruentata*), schoolmaster (*L*. *apodus*), lane snapper (*L*. *synagris*) and mahogany snapper (*L*. *mahogoni*), and *p* values from Mann-Whitney U tests of mean rank size.

FL distributions from the LPNR were compared for yellowtail snapper, schoolmaster, lane snapper, coney, graysby, hogfish, and mahogany snapper. Mean FL decreased significantly between periods for all species except lane snapper, which showed no significant change (Table 10, Fig 9). The largest relative decrease in FL was estimated for coney, which dropped by 20–25%, from 17.89 cm (±0.15 cm) to 13.77 cm (± 0.32 cm). Magnitudes of mean decrease, with standard error margins, varied among other taxa: 15–21% for graysby, 13–19% for hogfish, 7–13% for schoolmaster, 7–12% for mahogany snapper, and 3–9% for yellowtail snapper (Fig 9).

**Table 10:** LPNR size distribution test results.

Species	KS test	Mann-Whitney U
*L*. *apodus*	*p* = 0.15	*p* < 0.01
*O*. *chrysurus*	*p* = 0.09	*p* < 0.001
*L*. *maximus*	*p* < 0.001	*p* < 0.0001
*L*. *synagris*	*p* = 0.57	*p* = 0.36
*C*. *fulva*	*p* < 0.0001	*p* < 0.0001
*C*. *cruentata*	*p* < 0.0001	*p* < 0.0001
*L*. *mahogoni*	*p* < 0.05	*p* < 0.05

Summary of results from two-sample Kolmogorov-Smirnov (KS) tests of the equality of size distributions, and Mann-Whitney U tests for location effects in species FL distributions from the LPNR. Comparisons conducted between periods (2004–2006 vs. 2017).

## Discussion

Our results indicate a weak reserve effect in the MNTZ since 2005 as evidenced by increased densities and mean FL of some snapper and grouper species; however, the largest snapper and grouper (Nassau grouper, tiger grouper, yellowfin grouper, dog snapper) were rare and showed little or no sign of recovery. Multivariate density effects were driven by increases in smaller predatory species, although only one, the coney, increased significantly in mean univariate density. Decreased density and increased mean FL for red hind imply low recent recruitment. However, greater density of the largest individuals suggests greater adult survival, which may be attributable to no-take protection. Our data suggest that populations of commercially important species within the MNTZ will need substantially more time, and likely better enforcement, to clearly demonstrate if a reserve effect is leading their recovery, but slight positive changes after 14 years of protection are apparent. Results from the LPNR indicate density increases for some species are likely due to recent recruitment (e.g., *C*. *fulva*, *O*. *chrysurus*, *L*. *maximus*). Yet, decreases in the mean FL of other taxa (e.g., *C*. *cruentata*, *L*. *apodus*, *L*. *mahogoni*), with no density trends, is consistent with chronic fishing pressure shaping population structures.

We found increases in total density in the MNTZ across years owing chiefly to significant increases for coney. Size trends for the species show a recruitment pulse in 2009–2010 (Fig 8) with smaller mean size in 2010 relative to 2005, and a significant increase in density over that period. Coney abundance gains in this case are probably not directly related to NTZ protection given the species is of low interest in the commercial fishery, including at Mona [46,66]. That coney was the only species assessed to show a pronounced recruitment signal is more likely reflective of the species’ comparatively large spawning population in 2005, conferring a better probability of self-recruitment [36]. Alternatively, coney may have benefit indirectly from widespread coral mortality following the 2005 bleaching event, with a reported increase in damselfish prey [67], although this remains untested.

In comparison, three species from LPNR (*C*. *fulva*, *L*. *maximus*, and *O*. *chrysurus*) showed recent recruitment pulses (Table 8, Fig 9). The strongest recruitment signal was observed for *L*. *maximus)* despite having the smallest absolute and proportional abundance of sexually mature individuals in 2017 (n = 6 in 220 transects). Several explanations for this apparent incongruity are possible: (*i*) recent environmental conditions were especially favorable for self-recruitment thus improving rates of larval retention, settlement and/or survival and counteracting the limitations imposed by few spawning individuals [68–70]; (*ii*) the spawning population of *L*. *maximus* in the survey domain is larger than indicated because habitats containing greater densities of sexually mature individuals were not sampled; (*iii*) larval connectivity within a metapopulation on the Puerto Rican platform increased the odds of strong recruitment in the LPNR regardless of the local spawning population size [44]. Regarding point (*ii*), Pittman et al. (2010) surveyed a wider set of habitat types across the insular shelf and found similarly low numbers of sexually mature individuals (n = 23 in 1167 transects). In all likelihood, local production and larval-subsidy from nearby areas of the contiguous insular shelf dually contribute to strong recruitment events in *L*. *maximus* and other reef fishes of the LPNR [71,72]. Larval subsidy into the LPNR would help explain why two heavily fished species (*O*. *chrysurus* and *L*. *maximus*) [6,73] showed increased recruitment without significant trends in the densities or sizes of sexually mature individuals. In contrast, only the coney, a non-target species with the largest mean density assessed, showed substantial recruitment in the MNTZ. These results support the contention that population replenishment at Mona operates according to an entirely different, more restrictive, set of conditions than in the LPNR.

Out of the four species assessed from the MNTZ with sufficient sample sizes for individual analysis, red hind is by far the most prized by fishers and would therefore be expected to show the greatest response to no-take protection [73,74]. That the species increased in mean length but decreased in mean density since 2005 suggests low recent recruitment. Decreases in red hind density since 2005 at several sites (west 1, west 2, south 3, and south 4) (Figs 1, 3a and 3b), are congruent with a displacement of fishing effort following implementation of no-take regulations in 2004, which left parts of the insular shelf unprotected. In particular, decreased red hind density in the west zone (open to fishing until 2010), supports a concentration of fishing effort there between 2005 and 2010. Notably, this zone is located immediately adjacent to the island’s largest campground and only continually occupied settlement. The fact that density remains depressed 7 years after expansion of the NTZ and closure of the western fished areas supports the notion that red hind recruitment has been low in the intervening years. However, positive trends evidenced in the population structure include a significant change in mean length of + 8.0 cm across survey methods, and a proportional increase of the largest size classes (Fig 8, Table 9). These size trends approach a 9.5 cm increase in mean length reported from a US Virgin Islands spawning aggregation after 12 years of no-take protection [75], reflecting greater adult survival (a trend not observed in LPNR for any species). This larger average size for red hind is what offset the decline in density to keep biomass (and fecundity [76]) stable, thus maintaining the potential for future recruitment.

Larger sized grouper and snapper (*E*. *striatus*, *M*. *venenosa*, *M*. *tigris*, and *L*. *jocu*), remained rare at Mona, with 117 individuals sighted in 682 surveys conducted since 2005. Low numbers precluded any meaningful analyses of population trends to evaluate their recovery. However, reports from other regional coral reef studies and interviews with elder fishermen suggest that the snapper and grouper assemblage at Mona remains altered. Previously fished taxa at Mona such as the goliath grouper (*E*. *itajara*) and Nassau grouper (*E*. *striatus*) were either absent or virtually absent from surveys. Mean density of large groupers as a group was 42% less than estimated at Navassa Island, a similarly isolated, oceanic location west of Hispaniola [77]. Mean sighting frequency of this group (3.2%) was also substantially lower than reported in other, lightly fished or unfished Caribbean locales such as South Water Caye, Belize (21.0%), the Dry Tortugas, Florida (30.6%), or the Exuma Cays Land and Sea Park, Bahamas (8.8%) [78–80].

Despite increases in total fish density in the LPNR attributable to greater recruitment for some species, signs of fishing mortality effects on populations are also apparent in our data. Large-sized species of epinephelids and lutjanids were exceedingly rare in the LPNR with 85 individuals sighted in 1219 surveys conducted between 2004 and 2017, precluding species-specific analyses of population trends. However, compared to sighting frequencies collected in 1980–1981, most of these species are now markedly rarer [81]. In 1980 and 1981, Nassau grouper were seen at 34% of all coral reef sites; in 2017 sighting frequency was less than 1%. Red hind were observed at 64.7% of sites in 1980–1981; in 2017, sighting frequency for the species was 8.2%. During 1980–1981, tiger grouper (*M*. *tigris*) were seen at 17% of shelf-edge sites. In this study, not a single tiger grouper was observed between 2004 and 2017. While Kimmel [81] used a different survey methodology, precluding direct comparison of densities, the magnitude of declines suggests that overfishing continues in the LPNR, following island-wide trends [6].

The possibility exists that our study lacked sufficient power to detect changes in the population metrics of some species in both study sites. High and heterogeneous variance between years and periods emerged in the counts and sizes of multiple taxa and may have obscured true effects (Tables 3 and 6). One remedy would have been to explicitly consider habitat variables within analyses to better account for fine-scale population variability. Such an approach may have allowed us to address the influence of the 2005 mass bleaching event and subsequent loss of live coral cover [67, 82] in shaping current reef fish demographics in both MPAs. While there is reason to propose that an abrupt change in benthic cover could have produced bottom-up trophic effects, potentially altering the demographics and distribution of our focal species, fishing pressure (or the protection from) has been shown to better explain variance in populations of target species [83–85]. One particularly limiting aspect of our experimental design was the lack of a control site on the Mona insular platform by which to separate the effects of fishing from environmental factors. While impossible given the extent of the MNTZ, a nearby control site would have allowed for a more unequivocal assessment of recovery. A lack of seasonal replication in 2005 and 2010 samplings at Mona may have also limited our ability to distinguish seasonal effects from the effects of fishing prohibition. Despite sampling time (season) not being a significant factor in analysis, seasonal processes are well known to shape coral reef ecosystems and may have contributed unexplained variance to our results [70,86]. Another potentially confounding aspect of our study are differences in data collection between MPAs. However, since MPAs were not directly compared, with statistical comparisons *only* between temporal samplings within each MPA, we believe that differences in methodology did not substantively influence our conclusions.

Conversations with fishers, park rangers, and visitors to the island indicated a degree of ongoing fishing within the MNTZ boundaries, corroborating previous reports of non-compliance [46] and low effectiveness of law enforcement. While we are unable to infer the present extent of poaching, or what level would negate no-take benefits, even minimal levels of fishing could significantly extend recovery time or prevent it entirely [87,88]. Taking red hind as an example species, outcomes from other regional closures are informative. Marshak and Appeldoorn [89] found that seasonal MPA closures at three red hind spawning aggregations, initiated in 1996 off western Puerto Rico were ineffective in generating population recovery due to a combination of increased effort outside of MPA boundaries and closures, as well as non-compliance with closures, despite the population apparently receiving substantial recruitment subsidy from nearby sources [90]. Nemeth et al. [91] documented less-than-expected benefits from a protected aggregation in St. Croix, USVI, which they ascribed to poaching and harvest outside of the seasonal MPA boundaries. With this is mind, future assessments of the MNTZ should prioritize quantifying non-compliance to be able to separate the relative importance of recruitment isolation and fishing mortality in shaping recovery.

The lack of significant trends in the biomass and density of large snapper and grouper species in the MNTZ likely reflects slow recovery rates due in part to recruitment isolation. Larval connectivity within a metapopulation is known to be a strong determinant of recovery in marine fishery reserves [36]; well-connected populations have the potential to recover more quickly once fishing is prohibited [92]. Empirical estimates of dispersal distance collected regionally would help provide an indication of the importance of self-recruitment to population persistence at Mona. However, such estimates are uncommon, and nonexistent for our focal species. Looking farther afield, Almany et al. [93] calculated the dispersal kernel for an Indo-Pacific grouper (*Plectropomus areolatus*) showing that 95% of larvae dispersed less than 32.5 km from a spawning site. A similar study using genetic parentage analysis reported that the Spanish flag snapper (*Lutjanus carponotatus*) dispersed a maximum of 28 km from natal reefs in northeast Australia [94]. While not perfect proxies, these estimates suggest that the 44 km separating Mona Island from the nearest source of larvae would be a substantial barrier to demographic-shaping recruitment events. This is further emphasized by regional population connectivity studies of a jawfish [43], a goby [39], and a broadcast-spawning coral [42], all finding that the Mona Passage acts as a regional filter to dispersal for those species. While low demographic connectivity is evident, historical and contemporary fishing pressure have also almost certainly undercut the recovery potential of populations in the MNTZ [95, 96]. While our methods do not permit us to separate the effects of fishing pressure and recruitment isolation, results presented here imply that a combination of the two is responsible for the slow recovery of large predators. Other studies have estimated more than 20 years for predatory fish biomass to reach carrying capacity in high-compliance, tropical and sub-tropical marine reserves of mixed connectivity [10,97,98]. In one of the longest running marine reserve studies to date, Russ and Alcala [99] estimated 15–40 years for large reef predator biomass to reach carrying capacity in two nearshore Philippine reserves situated ca. 8 km from the mainland. It is reasonable to assume that those reserves would have higher rates of larval immigration, and thus faster recovery times than the MNTZ [36]. With these points in mind, 14 years has likely been insufficient for targeted fish populations at Mona to accrue gains in density and biomass. Additional decades of protection and better compliance with NTZ regulations are presumably required for recovery to be fully realized.

Evidence of slow recovery in the MNTZ presented here shows that it may be inappropriate to manage the reserve with future fishery yields in mind, as this will lead to undue expectations of its productive capacity. Given the narrow insular shelf with limited coral habitat and apparent infrequency of larval inflow, large-bodied demersal species at Mona likely cannot sustain more than low levels of harvest once stocks have recovered. Management emphasis for the Mona NTZ should instead be framed in the context of marine heritage conservation and biodiversity preservation, with the express benefit of supporting regional genetic connectivity between the eastern and western Caribbean.
